# Efficacy of mepolizumab for patients with severe asthma and eosinophilic chronic rhinosinusitis

**DOI:** 10.1186/s12890-019-0952-1

**Published:** 2019-10-12

**Authors:** Takanori Numata, Katsutoshi Nakayama, Hirofumi Utsumi, Kenji Kobayashi, Haruhiko Yanagisawa, Mitsuo Hashimoto, Shunsuke Minagawa, Takeo Ishikawa, Hiromichi Hara, Jun Araya, Kazuyoshi Kuwano

**Affiliations:** 1 Division of Respiratory diseases, Department of Internal Medicine, Jikei University School of Medicine, 3-25-8, Nishi-Shimbashi, Minato-ku, Tokyo, 105-8461 Japan; 2 Department of Respiratory Medicine, Akita University Graduate School of Medicine, 1-1-1 Hondo, Akita, 010-8543 Japan

**Keywords:** Asthma, Mepolizumab, Eosinophilic chronic rhinosinusitis, Predictive factor, *Staphylococcus* enterotoxin

## Abstract

**Background:**

Several major randomized control studies have demonstrated that mepolizumab, an anti-IL-5 monoclonal antibody, is effective for patients with severe eosinophilic asthma who show exacerbation or require systemic corticosteroid maintenance therapy. However, the predictive factors of the response to mepolizumab other than blood eosinophil count are unclear in clinical practice.

**Objective:**

To elucidate the predictive factors of the response to mepolizumab for patients with severe eosinophilic asthma.

**Methods:**

From July 2016 to December 2017, 28 patients with severe asthma received mepolizumab in our hospital. To determine the predictive factors, we retrospectively evaluated patient characteristics, comorbidities, biomarkers, pulmonary function, maintenance dose of systemic corticosteroids and number of exacerbations.

**Results:**

The response rate to mepolizumab treatment was 70% (19/27; one pregnant woman was excluded from analysis). Compared with 11 patients without eosinophilic chronic rhinosinusitis (ECRS), 16 patients with ECRS showed significantly improved systemic corticosteroid-sparing effects [− 71.3 ± 37.0% vs − 10.7 ± 20.1%, *P* = 0.006], change from baseline FeNO [− 19 ± 57 (%) vs 30 ± 77 (%), *P* = 0.023] and symptoms [14 patients (88%) vs five patients (45%), *P* = 0.033]. ECRS was identified as a predictive factor of the response to mepolizumab in a multivariate logistic regression analysis [odds ratio = 22.5, 95% CI (1.5–336), *P* = 0.024]. Of the eight patients previously administered omalizumab, five responded to mepolizumab. *Staphylococcus aureus* enterotoxin B IgE results were negative in 80% of responders (*P* = 0.14).

**Conclusion:**

Both groups showed improved symptom scores and a decreased number of exacerbations. Mepolizumab substantially improved the clinical variables of patients with eosinophilic asthma complicated with ECRS.

## Introduction

It is estimated that as many as 300 million people of all ages and ethnic backgrounds suffer from asthma [1]. Asthma is a common chronic respiratory disease that affects approximately 5–10% of Japanese adults [2, 3]. The prevalence of severe and uncontrolled asthma among adults with asthma has been reported to be 3 to 10% [4]. Severe asthma is associated with morbidity, mortality, and high healthcare costs [5]. Recently, severe asthma was classified into several clinical phenotypes that differ in severity and response to therapy [6]. Severe eosinophilic asthma is characterized by Th2 inflammation, and patients with severe eosinophilic asthma experience recurrent asthma exacerbations even when treated with high doses of inhaled corticosteroids (ICS) and controllers such as long-acting bronchodilators, leukotriene receptor antagonists and oral corticosteroids (OCS) [7]. Twenty to 25% of patients with difficult-to-control asthma have nasal polyps [8]. Furthermore, patients with asthma and high blood eosinophil counts have poor asthma control [9].

IL-5, which induces the proliferation, differentiation and migration of eosinophils, plays an important role in the pathogenesis of eosinophilic asthma [10]. Mepolizumab, a humanized monoclonal antibody against IL-5, selectively inhibits eosinophilic airway inflammation. Several major randomized control trials (RCTs) have demonstrated that this antibody reduces the number of eosinophils in both sputum and blood, resulting in reductions in exacerbations, improvements in pulmonary function and a glucocorticoid-sparing effect in patients with uncontrolled asthma and a peripheral blood eosinophil count ≥150/mm^3^ at baseline or ≥ 300/mm^3^ during the previous year [11–13]. Although previous reports have demonstrated the efficacy of this treatment in patients with severe eosinophilic asthma, patients who meet the criteria of eosinophil counts do not always respond to mepolizumab. Accordingly, the identification of predictive factors of a response is very important from a medical economic perspective. However, few reports have examined the predictive factors for mepolizumab; these have been limited to RCTs and their subgroup analyses [11, 14] and a cluster analysis of the response to mepolizumab [15]. The reported main distinctive features of patients with good response are blood eosinophilia ≥150/mm^3^, airway reversibility > 16.2% and a body mass index (BMI) ≥30 kg/m^2 15^. BMI has been reported to be lower in Japanese patients with asthma than in Caucasian ones [16]. To elucidate the clinical efficacy of mepolizumab in Japanese patients with severe asthma, we performed a single-center, retrospective study.

## Methods

### Subjects

From July 2016 to December 2017, 28 adult patients with severe uncontrolled asthma received mepolizumab injections (100 mg every 4 weeks) in our hospital. Severe asthma was defined according to the Global Initiative of Asthma (GINA) guidelines and required high doses of ICS plus at least one of the following additional controllers: long-acting beta-agonists (LABA), long-acting muscarinic antagonists (LAMA), leukotriene receptor antagonists (LTRA), xanthine derivative and daily OCS [5]. All patients with asthma were diagnosed by respiratory physicians based on past medical histories of asthma, typical symptoms (cough, wheezing, circadian variation and seasonal episodes) and pulmonary function compatible with asthma. Severity at baseline and during exacerbation was defined according to the GINA guidelines [5]. The present study was approved by the Ethical Committee of Jikei University (28–259 (8502)), and informed consent was obtained from all subjects. The patient inclusion criteria were as follows: 1. at least two exacerbations requiring OCS treatment in the previous year, and 2. a blood eosinophil count of at least 150/mm^3 12^.

We retrospectively examined the following patient characteristics and parameters at baseline and at the last follow-up: gender, age, underlying eosinophilic diseases, smoking status, body mass index, peripheral blood eosinophil count, serum IgE, serum *Staphylococcus aureus enterotoxin* (SE)-IgE level, FeNO, Asthma Control Test (ACT) score, pulmonary function test results [forced volume capacity (FVC), forced expiratory volume in one second (FEV_1_), FEV_1_/FVC, and %FEV_1_], the severity of asthma according to the GINA guidelines [5], history of previous omalizumab treatment, daily systemic corticosteroid maintenance therapy, the number of exacerbations of asthma symptoms requiring systemic corticosteroids, duration of asthma, number of injections and observation period.

The underlying eosinophilic diseases considered included eosinophilic chronic rhinosinusitis (ECRS), eosinophilic granulomatosis with polyangiitis and eosinophilic otitis media. The FeNO level was measured using a NIOX VERO™ analyzer (Aerocrine AB, Stockholm, Sweden) according to the American Thoracic Society/European Respiratory Society recommendations at a 50 mL/s flow rate [17]. Experienced technicians measured FeNO and remeasured if necessary. We recorded the presence of ECRS or chronic rhinosinusitis with nasal polyps (CRSwNP) as evaluated by computed tomography findings, peripheral blood eosinophil count and the presence of nasal polyps according to the Japanese Epidemiological Survey of Refractory Eosinophilic Chronic Rhinosinusitis (JESREC) study [18] or by pathological findings. We analyzed all patients and compared two groups: patients with ECRS (ECRS group) and those without (non-ECRS group). The levels of IgEs specific to SEs, *Staphylococcus aureus* enterotoxin A (SEA) and *Staphylococcus aureus* enterotoxin B (SEB), were measured with a fluorescence enzyme immunoassay (SRL, Tokyo). The lower limit of SE-IgE detection was 0.10 kU/L. Sensitization was defined as a specific IgE level greater than 0.10 kU/L.

### Evaluation of the response to mepolizumab

The mepolizumab response in all cases with inconclusive results were categorized as ineffective. For the remaining cases, we defined a response to mepolizumab as improvement in one or more of the following parameters: change in ACT from baseline to the last follow-up, change in the number of asthma exacerbations per year and reduction of the daily dose of OCSs. The minimal clinically relevant difference was defined as an ACT score of three points [19]. Systemic corticosteroid doses are shown as prednisone equivalents (mg).

### Statistical analysis

All statistical analyses were performed using StatView version 5 (SAS Institute Inc., Cary, NC, USA). All values are expressed as the mean ± standard deviation. A *P*-value of < 0.05 was considered statistically significant. The factors associated with patient characteristics were examined using Mann-Whitney *U* tests, Fisher’s exact tests, or Wilcoxon signed rank tests. Moreover, we reevaluated several primary outcomes by performing a post-hoc power analysis. Logistic regression analysis was performed to evaluate potential predictive factors (in a multivariate model), including age (≥ 65 years), gender (male), and other variables that achieved a value of *P* < 0.10 in the univariate models. The cut-off value of FeNO (≥ 50) was applied based on RCTs [11] and guidelines [20].

## Results

### Assessment of all patients

We excluded one pregnant woman who had received only one injection of mepolizumab from evaluation. The characteristics of the remaining 27 patients are shown in Table 1. Eight patients (30%) previously received omalizumab treatment. Among the 27 patients with severe asthma, 16 patients (59%) needed OSC maintenance therapy, and 16 patients (59%) suffered from ECRS as a comorbidity. The median number of mepolizumab injection was nine (range 2–17), and there was no significant difference between the ECRS and non-ECRS groups. The median duration of observation was 11 months (range 4–17), and there was a significant difference in observation duration between the two groups. Initial treatment at baseline is shown in Table 1. All patients received ICS/LABA, and 25 patients (93%) received high-dose ICS (≥ 800 μg, fluticasone propionate or equivalent). There was no significant difference regarding initial treatment between the ECRS and non-ECRS groups.

**Table 1:** patients’ characteristics at baseline

	all patients	ECRS (+) group	ECRS (−) group	*p* value between two groups
	*n* = 27	*n* = 16	*n* = 11	
gender (male), *n* (%)	8 (30)	5 (31)	3 (27)	0.99 < ^a^
age (year-old), (range)	56.3 (11.8), (35–79)	54.2 (11.3), (35–71)	59.3 (12.5), (36–79)	0.31^b^
duration of asthma (year), (range)	19.6 (11.9), (4–43)	23.5 (10.6), (8–43)	45.4 (22.6), (4–39)	0.020^b^
BMI(kg/m^2^)	22.8 (4.7)	22.1 (4.5)	23.9 (4.9)	0.26^b^
smoking (never / former / current), n	20 / 7 / 0	12 / 4 / 0	8 / 3 / 0	0.99 < ^a^
atopic / non atopic, n	21 / 6	15 / 1	6 / 5	0.027^a^
previous omalizumab, *n* (%)	8 (30)	6 (38)	2 (18)	0.40^a^
initial treatments use				
—ICS/LABA, n(%)	27 (100)	16 (100)	11 (100)	NA
—ICS dose(μg)^c^, mean (SD)	963 (222)	950 (137)	982 (316)	0.60
—LAMA, n(%)	13 (48)	5 (31)	8 (73)	0.054
—LTRA, n(%)	23 (85)	13 (81)	10 (91)	0.62
—xanthine derivative, n(%)	17 (63)	9 (56)	8 (73)	0.45
—maintenance therapy of OCS, *n* (%)	16 (59)	10 (63)	6 (55)	0.71^a^
—daily dose of OCS (mg)^d^, (range)	8.4 (5.6), (0.5–20)	7.0 (4.8), (0.5–15)	10.8 (6.5), (5–20)	0.29^b^
comorbidities, *n* (%)				
—ECRS	16 (59)	16 (100)	–	NA
—eosinophilic otitis media	9 (33)	9 (56)	0 (0)	NA
—EGPA	4 (15)	4 (25)	0 (0)	NA
number of injection, median (range)	9 (2–17)	9.5 (4–17)	7 (2–12)	0.17^b^
duration of observation (month), median (range)	11 (4–17)	12.5 (6–17)	10 (4–12)	0.014^b^

*ECRS* eosinophilic chronic rhinosinusitis, *BMI* body mass index, *NA* not available

*EGPA* eosinophilic granulomatous with polyangitis, *ICS* inhaled corticosteroid

*LABA* long-acting beta agonist, *LAMA* long-acting muscarinic antagonist

*LTRA* leukotriene receptor antagonist

Data presented as n(%) or mean (standard deviation), unless otherwise stated

^a^Fisher’s exact test, ^b^Mann-Whitney *U* test

^c^ICS doses are provided as fluticasone propionate equivalents (μg)

^d^Oral corticosteroid(OCS) doses are provided as prednisone equivalents (mg)

**Fig. 1:**
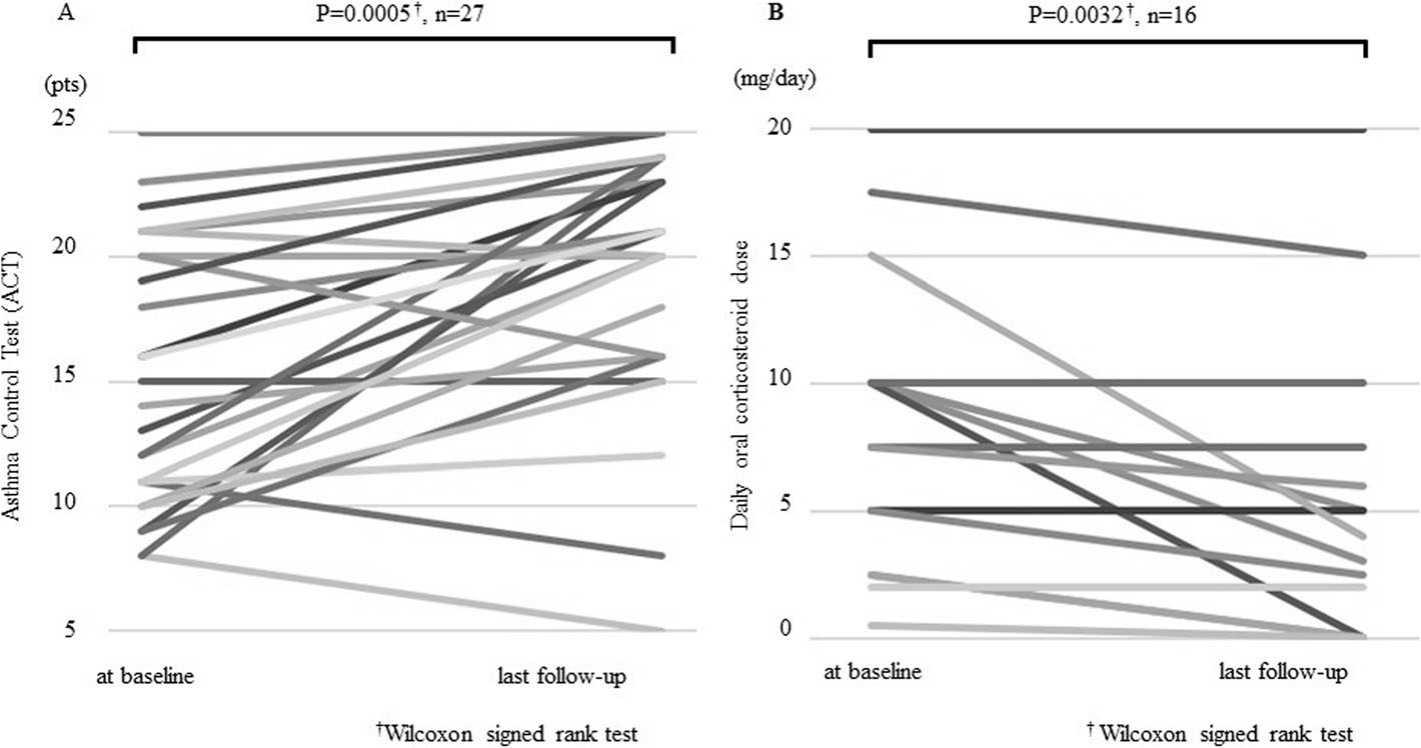
Efficacy of introduction of mepolizumab. **a** There was a significant change from baseline to the last follow-up in the Asthma Control Test (ACT) score (*P* = 0.0005, Wilcoxon signed rank test, *n* = 27). **b** There was a significant change from baseline to the last follow-up in the daily dose of oral corticosteroids (*P* = 0.0032, Wilcoxon signed rank test, *n* = 16)

The variables at baseline and the last follow-up of mepolizumab are shown in Table 2. We performed a post-hoc power analysis regarding the following primary outcomes in all patients, which are shown in Table 2: ACT score, daily dose of OCS and exacerbation. The power was over 0.80 except for the daily dose of OCS in all patients (0.62) (data not shown). The peripheral blood eosinophil count and serum IgE level were significantly decreased from baseline to the last follow-up, and the ACT score was significantly improved (Fig. 1a). The number of asthma exacerbations requiring systemic corticosteroids and the maintenance dose of OCS were significantly reduced after introducing mepolizumab (Fig. 1b). The mean BMI was 22.9 (kg/m^2^) in all patients. Of the 19 patients for whom SEA- and SEB-IgE tests were performed, 11 had negative SEB-IgE test results (57.9%) (data not shown). Furthermore, of the 13 patients who responded to mepolizumab, nine had negative SEB-IgE results (69.2%) (data not shown).

**Table 2:** change from baseline to last follow-up in asthma patients with or without ECRS

	all patients, *n* = 27			ECRS (+) group, *n* = 16			ECRS (−) group, *n* = 11			between two groups
	baseline	last follow-up	*p* value^a^	at baseline	at last follow-up	*p* value^a^	at baseline	at last follow-up	*p* value^a^	*p* value^‡^
blood eosinophil count(/mm^3^)	873 (1851)	71 (64)	< 0.0001	1219 (2353)	89 (74)	0.0004	338 (364)	44 (31)	0.005	0.0049
serum IgE (IU/ml)	485 (713)	305 (583)	0.016	440 (415)	261 (263)	0.0052	505 (992)	367 (873)	0.95	0.15^b^
FeNO (ppb)	62 (57)	44 (33)	0.056	84 (62)	53 (37)	0.021	32 (24)	32 (24)	0.53	0.007^b^
—Δ%FeNO (%), (range)	0.7 (69.2), (−68—180)			−19 (57), (−68—157)			30 (77), (−53—180)			0.023
ACT (pts)	15.2 (5.1)	19.5 (5.2)	0.0005	16.3 (5.5)	21.7 (4.8)	0.0023	13.6 (4.2)	16.4 (4.2)	0.066	0.16^b^
—ΔACT (pts), (range)	5.3 (4.5), (−4—16)			5.4 (5.4), (−3—16)			2.7 (4.4), (−4—9)			0.24
%FVC (%)	91.1 (14.8)	99.2 (17.5)	0.031	94.7 (14.9)	100.3 (12.9)	0.14	85.1 (13.2)	97.7 (23.8)	0.09	0.16^b^
%FEV_1_ (%)	83.3 (27.6)	83.9 (24.2)	0.62	77.4 (24.5)	79.0 (19.9)	0.36	92.1 (30.7)	91.5 (29.3)	0.44	0.35^b^
FEV_1_ / FVC (%)	71.1 (13.3)	68.9 (12.9)	0.32	66.6 (12.8)	65.2 (12.5)	0.78	77.8 (11.6)	74.6 (11.8)	0.11	0.026^b^
%PEF (%)	86.2 (28.2)	89.4 (23.6)	0.011	82.3 (30.8)	85.3 (24.0)	0.011	92.1 (24.1)	95.9 (22.7)	0.53	0.29^b^
V_50_/V_25_	3.5 (1.1)	3.5 (1.1)	0.61	3.5 (1.2)	3.1 (0.9)	0.81	3.5 (1.1)	4.1 (1.1)	0.26	0.78^b^
daily dose of OCS (mg)^§^, (range)	8.4 (5.6), (0.5—20) (*n* = 16)	5.0 (5.8), (0—20) (*n* = 16)	0.0032	7.0 (4.8), (0.5—15) (*n* = 10)	2.0 (2.4), (0—6) (*n* = 10)	0.008	10.8 (6.5), (5—20) (*n* = 6)	10.0 (6.5), (2.5—20) (*n* = 6)	0.16	0.29^b^
—ΔOCS (%), (range)	−48.6 (43.3), (−100—0)			−71.3 (37.0), (−100—0)			−10.7 (20.1), (−50—0)			0.006
exacerbation (/year), n	5.6 (4.9)	2.9 (3.7)	0.002	5.6 (5.6)	2.4 (3.3)	0.01	5.5 (3.9)	3.6 (4.2)	0.0498	0.77^b^
—Δexacerbation (%), (range)	−44.7 (61.4), (−100—125)			− 48.4 (62.6),(− 100—125)			−39.9 (62.6), (− 100—100)			0.82

*ECRS* eosinophilic chronic rhinosinusitis, *NA* not available, *ACT* Asthma Control Test Δ: change from baseline to the last follow-up Data presented as mean (standard deviation), unless otherwise stated. Blood eosinophil counts at last follow-up were examined at 3 months(median, range 1–6). Serum IgE tests at last follow-up were examined at 6 months(median, range 2–6)

FeNO tests at last folloow-up were examined at 9 months(median, range 1–12). Asthma Control Tests(ACT) score at last follow-up was examined at 6 months(median, range 2–12)

Pulmonary function tests at last follow-up were comducted at 4 months(median, range 2–10)

^a^Wilcoxon signed rank test ^‡^ Mann-Whitney *U* test ^§^ Oral corticosteroid(OCS) doses are provided as prednisone equivalents (mg). ^b^ at baseline

### Assessment of asthma patients with or without ECRS

**Fig. 2:**
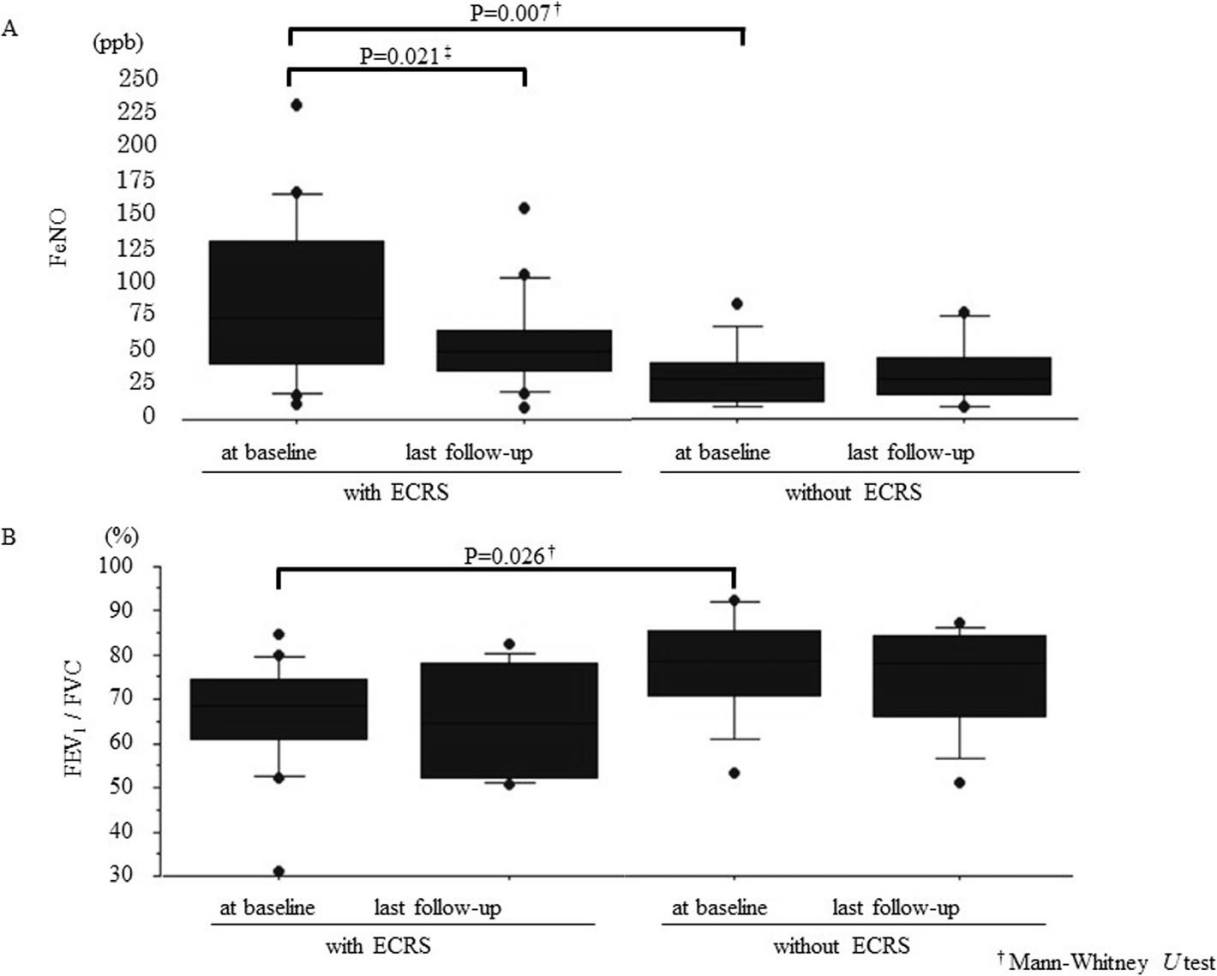
FeNO level and FEV_1_/FVC in the ECRS and non-ECRS groups. All results are expressed as individual data, and the boxes represent medians and interquartile ranges. The upper and lower whiskers represent the 90th and 10th percentiles, respectively. **a** FeNO level. **b** FEV_1_/FVC. ^†^Mann-Whitney *U* test, ^‡^Wilcoxon signed rank test

**Fig. 3:**
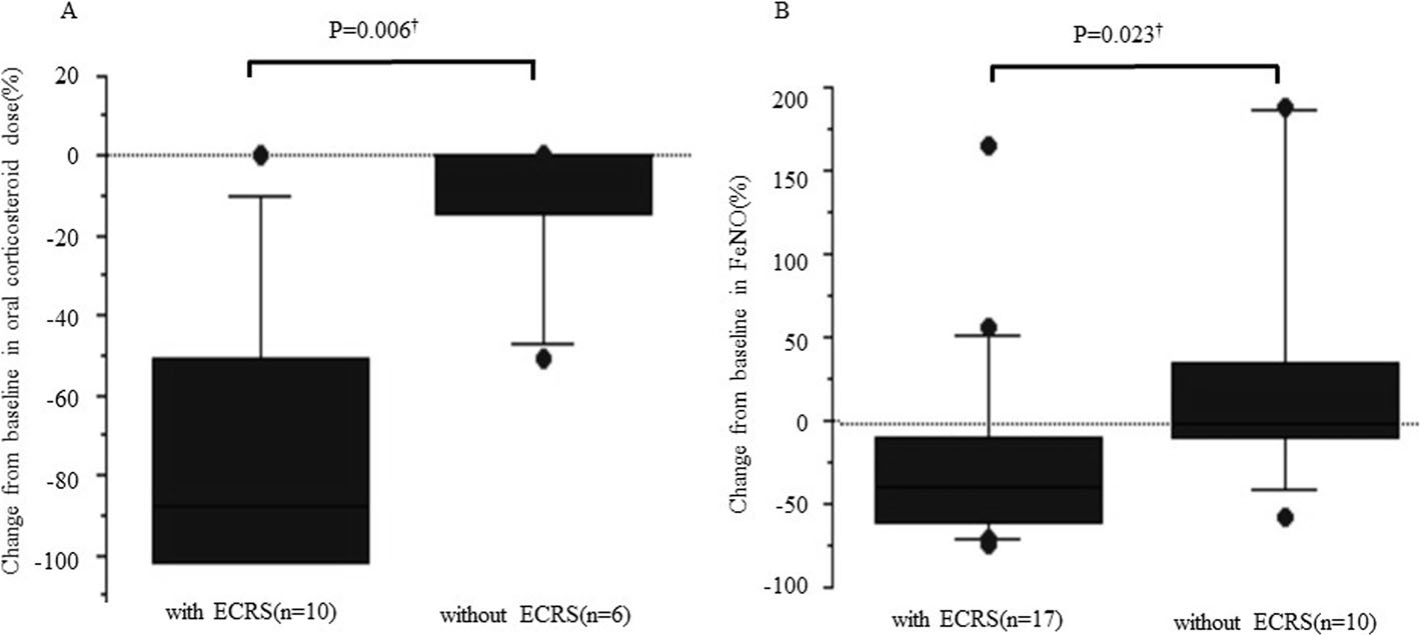
Comparison of clinical efficacy between the ECRS and non-ECRS groups. All results are expressed as individual data, and the boxes represent medians and interquartile ranges. The upper and lower whiskers represent the 90th and 10 percentiles, respectively. **a** Change from baseline in oral corticosteroid dose. **b** Change from baseline in FeNO. ^†^Mann-Whitney *U* test

We divided all 27 patients into two groups: and ECRS group (with ECRS) and a non-ECRS group (without ECRS). The characteristics of these patients are shown in Tables 1 and 2. Peripheral blood eosinophil count and FeNO level in the ECRS group were significantly higher than those in the non-ECRS group at baseline (Table 2 and Fig. 2a). Moreover, at baseline, FEV_1_/FVC was significantly lower in the ECRS group than in the non-ECRS group (Table 2 and Fig. 2b). The power was over 0.80 for the three primary outcomes in the ECRS group, however it was 0.31 for exacerbation in the non-ECRS group (data not shown). Between the ECRS and non-ECRS group, there was a significant difference in the percentage reduction of the daily dose of OCS [− 71.3 ± 37.0% and − 10.7 ± 20.1%, respectively, *P* = 0.006, Mann-Whitney *U* test] and in the change in FeNO level from baseline [− 19 ± 57% and 30 ± 77%, respectively, *P* = 0.023, Mann-Whitney *U* test] (Table 2, Fig. 3a and b). Of the16 patients in the ECRS group, 10 received daily OCS at baseline, and five patients stopped OCS treatment after the introduction of mepolizumab. There were two patients that exhibited an increased number of exacerbations despite improvement in ACT score. After considering these factors, we concluded that 14 patients (88%) with ECRS and five patients (45%) without ECRS showed clinical improvement (*P* = 0.033, Fisher’s exact test).

### Predictive factors

Among the 27 assessed patients, 16 patients (59%) showed a clinically relevant amelioration of ACT score (Table 1 and Fig. 1a). Furthermore, the maintenance dose of daily OCS (Table 2 and Fig. 1b) and the number of exacerbations were significantly reduced from 8.4 ± 5.6 to 5.0 ± 5.8 (mg/day) (*P* = 0.0032, Wilcoxon signed rank test) and from 5.6 ± 4.9 to 2.9 ± 3.7 (per year) (*P* = 0.002, Wilcoxon signed rank test), respectively, following the introduction of mepolizumab (Table 2). As mentioned above, we concluded that 19 patients (70%) showed clinical improvement. To identify predictive factors of mepolizumab response, we first analyzed each variable, such as patient background, comorbidities and biomarkers, by univariate logistic regression analysis (Table 3). We identified ECRS as a predictive factor of a mepolizumab response [odds ratio (OR) = 8.4, 95% CI (1.3–56), *P* = 0.028]. The variables that achieved a value of *P* < 0.10 in the univariate models included ECRS and maintenance OCS therapy. We then evaluated predictive factors including age (≥65 years), gender (male), ECRS and maintenance OCS therapy by multivariate logistic regression analysis. Only ECRS was identified as a predictive factor by multivariate logistic regression analysis [OR = 22.5, 95% CI (1.5–336), *P* = 0.024].

**Table 3:** predictive factors by logistic regression analysis

variables	effective	non effective	odds ratio(95% CI) (monovariate)	*P* value	odds ratio(95% CI) (multivariate)	*P* value
	*n* = 19	*n* = 8				
gender (male), *n* (%)	7 (37)	1 (13)	4.1 (0.4–40)	0.23	5.6 (0.28–115)	0.26
age (≥ 65 year-old), n(%)	6 (32)	2 (25)	1.4 (0.2–9.0)	0.73	1.2 (0.05–26.6)	0.92
duration of asthma (≥ 20 years), n(%)	11 (58)	4 (50)	1.4 (0.3–7.2)	0.71	–	–
BMI(kg/m^2^) ≥ 25, *n* (%)	6 (32)	2 (25)	1.4 (0.2–9.0)	0.73	–	–
atopic type, n(%)	16 (84)	5 (63)	3.2 (0.5–21)	0.23	–	–
blood eosinophil count≥150(/mm^3^)^a^, *n* (%)	18 (95)	7 (88)	2.6 (0.1–47)	0.54	–	–
FeNO≥50(ppb)^a^, *n* (%)	9 (47)	3 (38)	1.5 (0.3–8.1)	0.64	–	–
with maintenance therapy of OCS, *n* (%)	10 (59)	7 (75)	0.1 (0.01–1.3)	0.078	0.05 (0.001–1.5)	0.08
with ECRS as comorbidity, *n* (%)	14 (74)	2 (25)	8.4 (1.3–56)	0.028	22.5 (1.5–336)	0.024

*CI* confidence interval, *BMI* body mass index, *OCS* oral corticosteroid, *ECRS* eosinophilic chronic rhinosinusitis, ^a^ at baseline

### Previous treatment with omalizumab

Recently, a cluster analysis of patients with chronic rhinosinusitis with nasal polyp (CRSwNP) reported that both IL-5-positive clustering and SE-IgE-positive clustering were important factors for eosinophilic inflammation when asthma was included in the analysis as a comorbidity [21]. Among 27 patients, eight patients with inadequately controlled asthma despite omalizumab treatment were transitioned to mepolizumab treatment. Their ACT scores increased from 17.0 ± 4.6 to 21.1 ± 3.6 (*P* = 0.063, Wilcoxon signed rank test, data not shown), and all five patients (63%) who we assessed as effective cases had ECRS. All four patients whose serum SEB-IgE levels were negative (< 0.10 kU/L) tended to respond better to mepolizumab than to omalizumab (*P* = 0.14, Fisher’s exact test, Table 4). A significant difference was observed between the SEB-IgE-positive and SEB-IgE-negative groups in the change from baseline of the daily dose of OCSs [− 4.8 ± 8.2% vs − 64 ± 41%, respectively, *P* = 0.046, Mann-Whitney *U* test] (Table 4); with the power was greater than 0.80 according to the post-hoc power analysis (data not shown).

**Table 4:** association with SEB-IgE in the patients with previously omalizumab treatment (*n* = 8)

	SEB-IgE(+) *n* = 4	SEB-IgE(−) *n* = 4	*p* value
positive number with favours mepolizumab, n(%)	1 (25)	4 (100)	0.14^a^
ECRS as comorbidity, n(%)	2 (50)	4 (100)	0.43^a^
change from baseline in ACT score(pts)	4.5 (7.0)	3.8 (3.0)	0.77^b^
change from baseline in OCS dose^c^(%)	−4.8 (8.2)	−64 (41)	0.046^b^
change from baseline in exacerbation(%)	−8.8 (98)	−77 (42)	0.24^b^

*SEB-IgE Staphylococcus aureus* enterotoxin B specific immunoglobulin E antibody, *ECRS* eosinophilic chronic rhinosinusitis, *OMA* omalizumab, *ACT* Asthma Control Test, *OCS* oral corticosteroid

Data presented as mean (standard deviation), unless otherwise stated

^a^Fisher’s exact test

^b^Mann-Whitney *U* test

^c^OCS doses are provided as prednisone equivalents

There are three patients received maintenance OCS treatment in each groups

## Discussion

We demonstrated that mepolizumab substantially improved the clinical variables of patients with asthma complicated with ECRS. In the present study, we identified ECRS as a predictive factor based on multivariate logistic regression analysis. BMI was among the predictive factors of mepolizumab reported in the previous cluster analysis reported [15]; in the present study, we adjusted BMI to the Japanese obese standard (≥25 kg/m^2^). A recent US database study reported that the risk factors for asthma attack include blood eosinophilia, rhinitis and nasal polyps [22]. Patients with ECRS often have eosinophilia, and reduction of blood eosinophil count is important for the stability of asthma symptoms. Although endoscopic sinus surgery improves asthma symptom scores in patients with ECRS [23], the recurrence rate of severe ECRS is approximately 70% or higher, and repeat surgery is often required [24]. The deterioration of ECRS is related to asthmatic symptoms, and patients are often dependent on corticosteroids. We hypothesize that the anti-IL-5 antibody inhibits eosinophilic inflammation more effectively than corticosteroids not only in the lower airway but also in the upper airway. Because ACT scores may deteriorate if mepolizumab treatment reduces the daily dose of OCS, we evaluated multidisciplinary measures, such as ACT, the annual rate of asthma exacerbation and the OCS-sparing effect.

FeNO is useful for the diagnosis of asthma and for evaluating lower-airway inflammation. In the present study, FeNO levels in the ECRS group were higher than those in the non-ECRS group. This result is similar to that of a previous report [25]. Under eosinophilic inflammation, sinus tissue releases not only mediators and cytokines that directly induce inflammation of the lower airways but also chemotactic factors that recruit eosinophils from the bone marrow and circulation into the upper and lower airways [26, 27]. We observed that FeNO level significantly decreased from baseline in the ECRS group but not in the non-ECRS group. A potential reason for this result is that in the ECRS group, mepolizumab reduced not only the eosinophil counts in sinus tissue, but also eosinophilic airway inflammation. It is commonly reported that mepolizumab does not necessarily decrease FeNO level. Eosinophil-derived major basic protein (MBP) injures epithelial cells, which activates the innate immune system. Furthermore, group 2 innate lymphoid cells (ILC2s) and eosinophils release large amounts of IL-4, 5 and 13 [28, 29]. Hence, it is possible that anti-IL-5 therapy decreases peripheral blood and tissue eosinophils and indirectly inhibits the IL-4/IL-13 pathway, reducing serum IgE and FeNO levels.

Of the eight patients with previous omalizumab treatment in the present study, four patients (50%) had serum SEB-IgE levels lower than 0.10 kU/L; these patients responded to mepolizumab. On the other hand, three of the remaining four patients with positive serum SEB-IgE test results did not respond to mepolizumab. The prevalence of specific SE-IgE (≥0.10 kU/L) is approximately 40 to 75% in patients with asthma, and SE-IgE levels are associated with high IgE in eosinophilic asthma [30, 31]. Previous studies have suggested the potential involvement of SEB in the pathophysiology of not only eosinophilic asthma but also CRSwNP [32–35]. We occasionally observe patients with eosinophilic asthma who show slight or no response to mepolizumab in clinical practice. We suspect that such asthma patients are not IL-5-positive patients with CRSwNP/ECRS but are SE-IgE-positive patients. However, the number of patients with ECRS was small in the present study. Hence, additional data are needed to elucidate the usefulness of serum SEB-IgE in predicting a response to mepolizumab in patients with severe asthma and ECRS.

There are several limitations to the present study. One limitation is that it is a small, single-center, retrospective study. To increase the reliability of the statistical analysis, a power analysis was warranted. Thus, we performed a post-hoc power analysis regarding the primary outcomes in every group. A higher number of patients and a prolonged follow-up period are needed for more accurate evaluations. A second is that the limited induction of biologic therapy due to high cost may have resulted in selection bias. Accordingly, data collection and analysis in clinical studies that are distinct from major RCTs are important. In the future, detailed assessments of comorbidities in patients with asthma will be important for clinical practice.

In conclusion, the results of the present study showed that the improvement in the asthmatic symptoms of patients with ECRS was stronger than that of patients without ECRS. This information may be useful for managing the introduction of biologic treatments. The SEB-IgE test might be helpful for selecting mepolizumab or omalizumab therapy for patients with severe asthma.

## Data Availability

The datasets used and/or analyzed during the current study are available from the corresponding author on reasonable request***.***

## Abbreviations

ACT: Asthma Control Test
CRSwNP: Chronic rhinosinusitis with nasal polyp
ECRS: Eosinophilic chronic rhinosinusitis
GINA: Global Initiative of Asthma
OCS: Oral corticosteroids
RCT: Randomized control trial
SE-IgE: *Staphylococcus aureus* specific IgE
